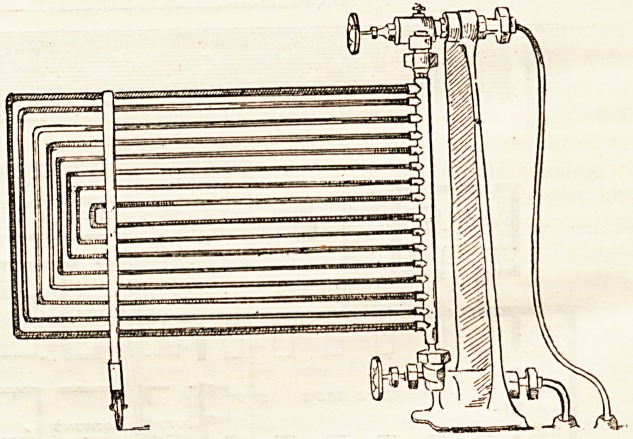# Practical Departments

**Published:** 1895-08-17

**Authors:** 


### PRACTICAL DEPARTMENTS.

### NEW OPERATING THEATRE, THE MIDDLESEX HOSPITAL.

The new operating theatre at the Middlesex Hospital, which has only quite recently been finished, is a triumph of modern science in all its arrangements, and well worth a visit from all and any who are interested in the newest hospital developments of to-day.

As an experiment in artificial heating and ventilation alone its success will be watched with interest; we believe we are right in saying it is the first theatre, in any London hospital, which has been treated exclusively by these means.

The new theatre is approached by a long passage, floored and walled, like every part of the new building, with particularly pretty tiles. And here let us remark that the wisdom of choosing artistic colours for such purposes seems to us to be undeniable, and the improvement in these details in modern buildings, and the thought which is nowadays manifestly bestowed upon their due carrying out, bears good comparison with the ways of the past. On the left hand side of the corridor are surgeons' rooms, &c., with an operating-room for minor operations. This is a good size, splendidly lighted, and fitted with every possible appliance. The large theatre will accommodate considerably more than a hundred students; but before commenting upon its fittings it will be better to give a general idea of the system upon which it is ventilated, from information which Messrs. J. Slater and Co., of High Holborn, who have carried out the work, have kindly supplied. The problem to be solved, in arranging for the most approved method of ventilating the new theatre, was how best to give a large quantity of pure warm air in winter and cooled air in summer, with complete control over temperature and quantity at all times, and freedom from draughts, the place being none too large for the number of students to be accommodated. The air, then, is first drawn through screens moistened with antiseptic spray, and driven from thence through beds of cotton wool into a large cylindrical container, which equalises the pressure; from the container it is carried by a system of iron pipes to the nozzles or air jets in various inlet or outlet tubes. Compressed air is the motive power, and the air is forced out as well as forced in to the several places required. A gas engine is used to work the air compressor. When air is required in any room the jets or tubes into and out are turned on by means of a valve, when the streams of compressed air issuing from the nozzles induce a strong current of air in the enclosing tubes into or out of the room, as the case may be. The air can be kept at any temperature up to 70° Fahrenheit on the coldest winter day, and can be altered from cold to warm, or the reverse, in a few minutes. Not the slightest feeling of draught can be detected in theatre or rooms when the air is passing through at a rate equal to a complete change ten times per hour. Thus economy is practised, for the compressed air is only turned on in such room or rooms as may happen to be in use at one time. The pipe chamber, in which the warming of the air is accomplished, is built of glazed brick, and the coils of pipes are made in large leaves like gates (on the same principle as the coils in the theatre itself, of which we give an illustration), and hung to fold open on each other in such a way as to admit of their being, even while in actual use, completely brushed or washed, and thus perfectly cleansed. The cleansing and purification of the galvanised iron tubes from air chamber to theatre and the other rooms, and from the same into the open air, can also be accomplished easily and with little expense. The boiler supplying the hot water in the wrought-iron coils in the warming chamber is fixed in an adjoining chamber.

The objections to artificial ventilation in hospitals generally naturally do not apply in such a case as that under consideration. The success of operations is now known to so largely depend upon atmospheric conditions that any means by which the purity and even temperature of the air can be secured must tend to the saving of life and amelioration of suffering, and as so doing must be welcomed. The work has been most excellently carried out in this instance by the architect, Mr. Keith Young, ably seconded by the resident medical officer, Mr. Farden, whose interest in the gradual perfecting of this model theatre has been great.

Our illustration shows the form of hot-water coils used. Those in the theatres are of brass, and, as will be seen, can be moved easily on castors for cleaning and better diffusion of heat. Sketches of some of the other fittings we hope to give in a future issue.

(*To be continued.*)

## Figures and Tables

**Figure f1:**